# Baicalein inhibits SARS-CoV-2/VSV replication with interfering mitochondrial oxidative phosphorylation in a mPTP dependent manner

**DOI:** 10.1038/s41392-020-00353-x

**Published:** 2020-11-13

**Authors:** Shichao Huang, Yu’e Liu, Yanan Zhang, Ru Zhang, ChengJie Zhu, Lihong Fan, Gang Pei, Bo Zhang, Yufeng Shi

**Affiliations:** 1 Tongji University Cancer Center, Shanghai Tenth People’s Hospital of Tongji University, School of Medicine, School of Life Sciences and Technology, Tongji University, Shanghai, 200092 China; 2 State Key Laboratory of Cell Biology, CAS Center for Excellence in Molecular Cell Science, Institute of Biochemistry and Cell Biology, Chinese Academy of Sciences, University of Chinese Academy of Sciences, Shanghai, China; 3 Key Laboratory of Special Pathogens and Biosafety, Wuhan Institute of Virology, Center for Biosafety MegaScience, Chinese Academy of Sciences, 430071 Wuhan, China; 4 Shanghai Key Laboratory of Signaling and Disease Research, Collaborative Innovation Center for Brain Science, School of Life Sciences and Technology, Tongji University, Shanghai, China; 5 Center for Brain and Spinal Cord Research, School of Medicine, Tongji University, 200092 Shanghai, China

**Keywords:** Target identification, Infectious diseases, Microbiology

**Dear Editor,**

The ongoing outbreak of pneumonia (coronavirus disease 2019, COVID-19) caused by severe acute respiratory syndrome coronavirus-2 (SARS-CoV-2) has become a global health emergency. *Scutellaria Baicalensis* Georgi extract, a Traditional Chinese Medicine (TCM) widely used for treating infection of multiple different viruses,^1^ also shows beneficial effects when treating COVID-19. However, underline mechanism for broad antiviral activity of *Scutellaria B*. extract remains elusive, which limits its further application.

We first tested the effect of Baicalein (Fig. 1a) and its analogy Baicalin (Supplementary Fig. S1a), two key components from *Scutellaria B*. extract, on replication of SARS-CoV-2 and VSV, two viruses have very different genomes but can be amplified in the same Vero E6 cells. SARS-CoV-2 causes a severe acute respiratory syndrome and should be handled in laboratories with biosafety level 3 or higher, while VSV associated disease in human is generally mild and multiple tools are available, which allows more detailed mechanistic studies in less restricted laboratories. Indeed, we use VSV-GFP, a recombinant VSV mutated with M51 protein and tagged with GFP in this study.^2^

**Fig. 1:**
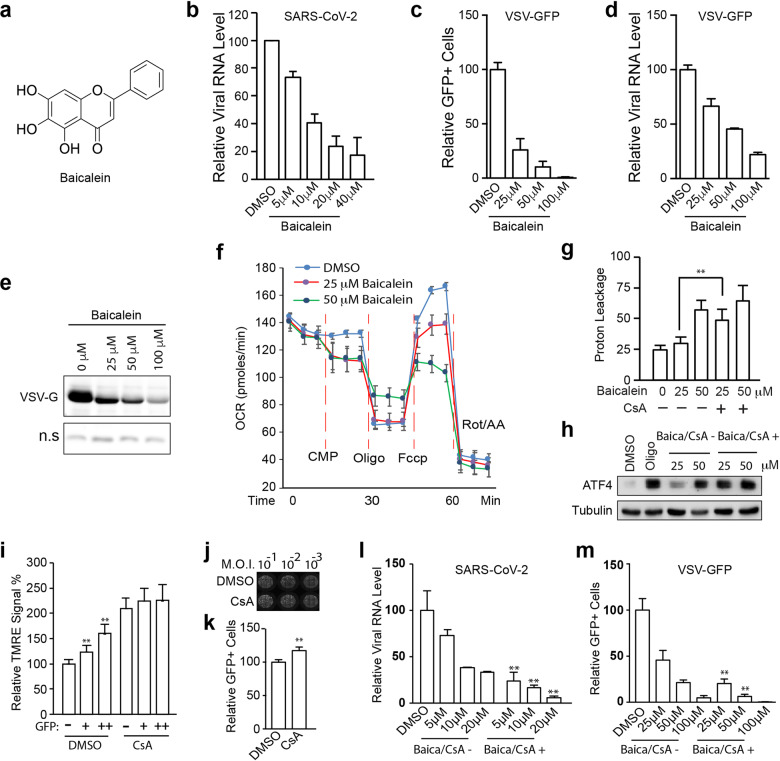
Baicalein interferes mitochondrial OXPHOS in a mPTP dependent manner and inhibits replication of SARS-CoV-2/VSV. **a** Baicalein structure. **b** RT-qPCR assays for SARS-CoV-2 RNA amplification in medium of Vero E6 cells show Baicalein inhibits SARS-CoV-2 replication. Mean ± sd; *n* = 3. **c** quantification of images (as of Fig. S1d) for VSV-GFP replication at different M.O.I. in the presence of increasing dose of Baicalein. **d** RT-qPCR assays show Baicalein dose dependently inhibits VSV RNA amplification in Vero E6 cells. Mean ± sd; *n* = 3. **e** Western blot shows Baicalein dose dependently blocks VSV-G protein expression in VSV-GFP infected Vero E6 cells. **f** Graph shows OCR measured by seahorse analyzer. OCR inhibition of Vero E6 cells by different dose of Baicalein (18 min), Oligomycin A (Oligo, 36 min) Fccp (54 min) and Rotenone/Antimycin A (Rot/AA, 72 min). *n* = 3; mean ± s.e.m. **g** Quantification of relative proton leakage from seahorse analyzer data as in **f**. **h** Western blot shows enhanced induction of ATF4 by Baicalein in the presence of mPTP inhibitor, CsA. **i** Quantification of flow cytometry data for TMRE signal (as in Supplementary Fig. S4a) indicates increased TMRE signal by VSV-GFP infection in Vero E6 cells. No significant difference of TMRE signal between VSV-GFP infected or none infected cells in the presence of mPTP inhibitor, CsA. **j** Representative image for VSV–GFP replication at different M.O.I. in the presence of CsA or not. **k** Quantification of **j**. **l** RT-qPCR assays for SARS-CoV-2 RNA amplification in medium of Vero E6 cells treated as indicated. Mean ± SD; *n* = 3. **m** Quantification of images (as in Fig. S4f) for VSV-GFP replication in Vero E6 cells treated as indicated. MOI multiplicity of infection; *p < 0.05; **p < 0.01

As shown in Supplementary Fig. S1b and S1c, Baicalein exerts a limited cytotoxic effect on Vero E6 cells at concentration of 100 μM or below, however, under the same conditions this small molecule potently inhibits replication of SARS-CoV-2 and VSV with IC50s around 10 and 15 μM, respectively (Fig. 1b, c and Supplementary Fig. S1d). Baicalein and Baicalin are both flavone molecules, Baicalin also shows minimum toxicity on Vero E6 cells (Supplementary Fig. S1b), but inhibits VSV replication with a higher IC50 (around 75 μM) comparing to that of Baicalein (Supplementary Fig. S1e,S1f), thus we mainly focus on Baicalein in following studies. Baicalein inhibits amplification of VSV genome RNA and expression of VSV glycoprotein G (VSV-G) at comparable IC50s indicating this small molecule might not target certain step of viral life cycle, but more likely interferes a virus–host interaction (Fig. 1d, e). Recently, chloroquine has been approved for treating COVID-19 in several countries, we found comparable potency of chloroquine and Baicalein in terms of their antiviral activities (Fig. 1c and Supplementary Fig. S1g, S1h). On the contrary, liquiritigenin, a TCM never shows antiviral activity, has no effect on VSV production (Supplementary Fig. S1i, S1j).

We identified Baicalein as an oxygen consumption inhibitor in a large-scale compound screen. The screen result is then verified and data further shows the IC50 for inhibition of oxygen consumption rate (OCR) by Baicalein is around 20 μM (Supplementary Fig. S2a). As about 95% of oxygen cell taking is consumed by OXPHOS, a central pathway for mitochondrial function governing mitochondrial membrane potential (MMP) and many others, a reduced OCR suggested an impaired OXPHOS by Baicalein. Consistently, Baicalein treatment causes a quick and robust decrease of MMP reflected by tetramethylrhodamine ethyl ester (TMRE) staining (Supplementary Fig. S2b). Detailed analysis further reveals a quick OXPHOS inhibition (within 6 min after Baicalein treatment) reflected by acute OCR inhibition, an instant glycolysis upregulation, a significant increase of mitochondrial membrane leakage and a suppressed cellular ATP level (Fig. 1f, g and Supplementary Fig. S2c, S2d, S2e). Mitochondrial OXPHOS inhibition could lead to an upregulation of ATF4, a transcription factor activated when cells are undergoing mitochondrial stress. Similar to known OXPHOS inhibitors (Rotenone for complex I, Antimycin A for complex III, and Oligomycin A for complex V in OXPHOS pathway), Baicalein dose dependently induces ATF4 expression (Supplementary Fig. S2f). However, under same conditions, Baicalein exerts minimum effect on functions of endoplasmic reticulum (ER) and peroxisomes reflected by a lacking of significant alternations in gene transcriptions, responsible for suppression of ER or peroxisome function (Supplementary Fig. S3a, S3b). Taking together, these data thus suggest Baicalein treatment quickly and robustly interferes mitochondrial OXPHOS, but not function of ER or peroxisomes.

Mitochondria exist in all eukaryotic cells, and classic mitochondrial OXPHOS inhibitors are often too toxic to be used in human or animals. Recent studies indicate animals are tolerable with conditional OXPHOS inhibitors, such as those whose activity in OXPHOS inhibition is depended on activity of mPTP, a recently defined OXPHOS component playing critical roles in MMP regulation. Application of Baicalein in human is safe, we thus check whether Baicalein OXPHOS inhibition can be moderated by blunting mPTP. Although Baicalein induced OCR inhibition is not affected by cotreatment of cyclosporine A (CsA), a classical mPTP inhibitor (Supplementary Fig. S2d), CsA significantly increases Baicalein induced proton leakage (Fig. 1g). Consistently, mPTP inhibition greatly enhances Baicalein induced ATF4 expression (Fig. 1h) suggesting Baicalein-mediated OXPHOS interference is reversely associated with mPTP activity.

Function of central metabolic pathways such as OXPHOS and mPTP are frequently altered by viruses for providing energy, biosynthetic resource, or evading immune surveillance to facilitate their production.^3,4^ We then check the role of virus on mitochondrial function in our system. VSV infection significantly increases the MMP in host cells (Supplementary Fig. S4a and Fig. 1i). This upregulated MMP is likely resulted from blunted mPTP by viral infection as no significant difference retains between infected and noninfected cells after cells are treated with mPTP inhibitor CsA (Fig. 1i). Similar results were recaptured in another VSV producing cells, HEK293 cells (Supplementary Fig. S4b), which implies virus interferes mPTP activity for its amplification.^3^ In line with this, mPTP inhibition by CsA significantly increases VSV replication (Fig. 1j, k).

OXPHOS has been purposed as a great target for treating viral infection.^3,4^ Consistently, treatments of Baicalein as well as other known OXPHOS inhibitors potently attenuate VSV production (Supplementary Fig. S4c–e). Baicalein OXPHOS inhibition is reversibly associated with mPTP activity (Fig. 1g, h). Consistently, Baicalein more potently inhibits SARS-CoV-2 and VSV replication in the presence of mPTP inhibitor CsA (Fig. 1l, m and Supplementary Fig. S4f). Recently, a small molecule Gboxin has been identified as an mPTP dependent OXPHOS inhibitor.^5^ Similarly, Gboxin antiviral activity is also greatly enhanced when mPTP is blunted (Supplementary Fig. S4g, S4h). On the contrary, Rotenone and Antimycin A do not rely on mPTP activity for their OXPHOS blockage, and mPTP inhibition does not further enhance their antiviral activities (Fig. S4i, S4j). In fact, CsA treatment partially rescues these compounds’ antiviral effect likely due to enhanced viral replication when mPTP is blunted (Supplementary Fig. S4i, S4j). As reported Baicalein treatment reduces intercellular ROS, a critical byproduct for OXPHOS, we checked whether Baicalein antiviral activity is associated with its activity in ROS clearance. However, data in Fig. S4k, S4l shows NAC, a well-established ROS scavenger, neither exerts any OXPHOS inhibition activity nor any antiviral activity, suggesting Baicalein’s antiviral activity is unlikely through ROS clearance.

This study thus shows Baicalein, a key component in TCM *Scutellaria B*. potently inhibits replication of SARS-CoV-2 and VSV. Mechanistically, Baicalein inhibits mitochondrial OXPHOS, and this inhibition is reversibly associated with mPTP activity in host cells. Viruses alter mitochondrial metabolism via blunting mPTP to facilitate their production, and OXPHOS suppression attenuates viral replication (Supplementary Fig. S5). Thus, our study not only for the first time provides clear evidences showing quick and robust OXPHOS inhibition by Baicalein, but also discovered a novel model of action for antiviral drug development, which is targeting mitochondrial OXPHOS/mPTP, a node of virus–host interaction.

## Supplementary information

Supplemental material
