# Supplementary material for: Baicalein inhibits SARS-CoV-2/VSV replication with interfering mitochondrial oxidative phosphorylation in a mPTP dependent manner
Source: Signal Transduct Target Ther. 2020 Nov 13;5:266. doi: 10.1038/s41392-020-00353-x (PMC7662024; doi:10.1038/s41392-020-00353-x)
Supplement: Supplementary file 1 — Supplemental material [file 41392_2020_353_MOESM1_ESM.pdf]

Supplementary Materials for

Baicalein inhibits SARS-CoV-2/VSV replication with interfering mitochondrial oxidative phosphorylation in a mPTP dependent manner

Shichao Huang, Yu'e Liu, Yanan Zhang, Ru Zhang, ChengJie Zhu, Lihong Fan, Gang Pei, Bo Zhang, Yufeng Shi

Correspondence to: Shichao Huang (huangshichao@sibcb.ac.cn), Bo Zhang (zhang-bo@wh.iov.cn) and Yufeng Shi (yshi@tongji.edu.cn).

This PDF file includes:

Materials and methods

Figures. S1 to S5

Materials and methods

Cell culture and virus

The SARS-CoV-2 virus (IVCAS 6.7512) was propagated and titrated in Vero E6 cell lines (ATCC® CRL-1586™) and HEK293 cells. Vero E6 and HEK293 cells were cultured in Dulbecco's modified Eagle's medium (DMEM; Invitrogen, Germany) containing 10% fetal bovine serum (FBS), 100 U/mL of penicillin and 100 μg/mL of streptomycin at 37℃ with 5% CO2. Viral titrations were performed with 10-fold serial dilutions in Vero E6 cells using plaque assay[1]. Three days post infection, plaques were calculated. VSV-GFP virus was amplified in the same Vero E6 cells and was used with multiplicity of infection (MOI) as indicated in figures.

Antiviral assay

Vero E6 cells were seeded into 24-well plates at a density of 8×10^4 cells per well one day before the assay. Then, cells were infected with SARS-CoV-2 at a MOI of 0.01 in the presence of different drugs as indicated. DMSO was used as controls. Efficacies were evaluated by quantification of viral RNA copy numbers in cell supernatant via quantitative real-time RT-PCR (qRT-PCR) and confirmed with visualization of virus nucleoprotein (NP) expression with immunofluorescence microscopy 24 h post infection (h.p.i.). For evaluating effect of different compounds on VSV-GFP replication, cells were seeded onto 96-well plates. On the following day, cells were treated with compounds for 2 hours before adding virus, and images were taken 8-10 hours later. Fluorescent images were taken 16 hours post infection using Zeiss observer Z1 microscope. ImageJ software was applied to quantify the number of GFP-positive (virus-infected) cells.

Antibodies and western blotting

After 16 hour-infection, Vero E6 cells were lysed with Laemmli's sample buffer. Then cell lysate was resolved by SDS-PAGE. Rabbit antibodies against VSV-G (1:1000, Sigma-Aldrich), and the peroxidase-conjugated, subtype-specific antibody (1:5000, Abmart) were used to detect viral protein. For cleaved caspase-3 detection, Rabbit antibodies against cleaved caspase-3 (1:1000, Cell Signaling Technology), and the peroxidase-conjugated, sub-type-specific antibody (1:5000, Abmart) were used. For ATF4 detection, cells are treated with compounds as indicated in figure for 6 hours, and then lysis in LDS Sample Buffer (Thermo). Antibodies used are anti-ATF4 (Santa Cruz, sc-200; C20; lot: #1914) and anti-tubulin (Sigma T9026). The blots were revealed by enhanced chemiluminescent detection (BioRad).

Real-time RT-qPCR

For checking SARS-CoV-2, RNA was extracted from cell supernatant using QIAamp viral RNA mini kit （52906， Qiagen） following the manufacturer's protocol. RT-qPCR were performed using Luna® Universal Probe One-Step RT-PCR Kit (E3006). Average values from duplicates of each gene were used to calculate the viral genomic copies.

RBD-qF1: 5'- CAATGGTTTAACAGGCACAGG-3'

RBD-qR1: 5'- CTCAAGTGTCTGTGGATCACG-3'

Probe: ACAGCATCAGTAGTGTCAGCAATGTCTC

For VSV, after 16 hour-infection, RNA was extracted from Vero E6 cells using TRI reagent (Sigma-Aldrich) following the manufacturer's protocol. The reverse transcription was performed using PrimeScript RT Master Mix (Takara). Then the cDNAs were used as template for qPCR. The primer sequences are as follows:

VSV gRNA-Forward: TTGGCAAGTATGCTAAGTCAG

VSV gRNA-Reverse: AGGACTTGAGATACTCACGAA

For RT-qPCR primer sequences of transcripts for genes critical for ER and peroxisome function:

ATF3-F: CCTCTGCGCTGGAATCAGTC;

ATF3-R: TTCTTTCTCGTCGCCTCTTTT;

CHOP-F: GGAAACAGAGTGGTCATTCCC

CHOP-R: CTGCTTGAGCCGTTCATTCTC

GRP78-F: CCACCAGGAAGATTGGCATTG

GRP78-R: CTTGCTGTAGGTCAGGCACT

GORASP2-F: AGGTGGAATCAAATTCTCCTGC

GORASP2-R: TCATGTGTTTCGATAAGGCTGAA

ARCN1-F: TGAAATTGTCGCACTGGGATAC

ARCN1-R: CTGACGGCTCTGAACACCTTC

ATF6a-F: TCCTCGGTCAGTGGACTCTTA

ATF6a-R: CTTGGGCTGAATTGAAGGTTTTG

PEX3-F: TGCTTCCAACACTGAGAGAGG

PEX3-R: AGAACAACCAGCATACAGGTACT

PEX11b-F: AGAAACAGATTCGACAACTGGAG

PEX11b-R: TGATAGGTGAACAGCTCTTTTGG

PEX19-F: AAAATGCCACTGACCTTCAGAA

PEX19-R: GTGATGGGTACAGCACATCCT

PEX7-F: TTGATGTGACTTGGAGTGAGAAC

PEX7-R: CCCACAATTTGACAGTTTGATCC

MFF-F: ACTGAAGGCATTAGTCAGCGA

MFF-R: TCCTGCTACAACAATCCTCTCC

FIS1-F: AGCGGGATTACGTCTTCTACC

FIS1-R: CATGCCCACGAGTCCATCTTT

Oxygen Consumption Rate (OCR)

OCR in Fig. S2a was performed with kit (MX-200-4, Agilent) following manufacturer's protocol, and 6 x 10^5 cells per well in 96-well plate were used. OCR in other figures was measured by Seahorse Bioscience instrument (XF24/XF96, Agilent) with 80-90% confluent cells according to the manufacturer's protocol. Briefly, on the day following cell seeding, cells were equilibrated for 1 hour in a 37°C incubator lacking CO2. Oxygen concentration in media was measured at basal conditions and after sequential addition of compounds as indicated in corresponding figure legends. Concentration of compounds used were: oligomycin A (1 μM); Fccp (1 μM); rotenone (1 μM); and a mixture of rotenone (500 nM) and antimycin A (500 nM). A minimum of three wells were utilized per condition to calculate OCR.

Mitochondrial Membrane Potential Measurement

TMRE mitochondrial membrane potential assay kit was purchased from Abcam (113852) and the assay was performed according to the manufacturer's protocol. For measuring mitochondrial membrane potential by flow cytometry, cells were pre-incubated with compound indicated in corresponding figures for 10 minutes and then incubated for an additional 20 minutes with TMRE (200 nM) before analysis in flow cytometry.

Cell viability assay

The cell viability of Vero E6 cells was evaluated using CCK-8 assay. Vero cells were plated in 96-well plates at a density of 1 x 10^4 cells per well. 24 hours later, cells were treated with

compounds for another 24 hours. Then 10 µl CCK-8 reagent (Beyotime) was added to each well and incubated for 1 hour in cell incubator. The optical density values at 450 nm were measured with a microplate reader (Multiscan FC, Thermo).

Cellular ATP level measurement

Vero cells were seeded in 96 well plates. Following overnight incubation, cells were treated with Baicalein for 6 hours at concentrations as indicated in the corresponding figure legends and then total cellular ATP level was measured by Cell Titer Glo® (Promega) assay.

Statistical Analysis and reproducibility.

Representative results from at least three experiments are shown for every figure except those specified in the figure legends. Data are presented as mean ± s.d. Statistical analysis was performed using software GraphPad Prism 7.0a. Statistical significance with p value was determined with the method used as indicated in corresponding figure legend.

Supplementary Figure 1

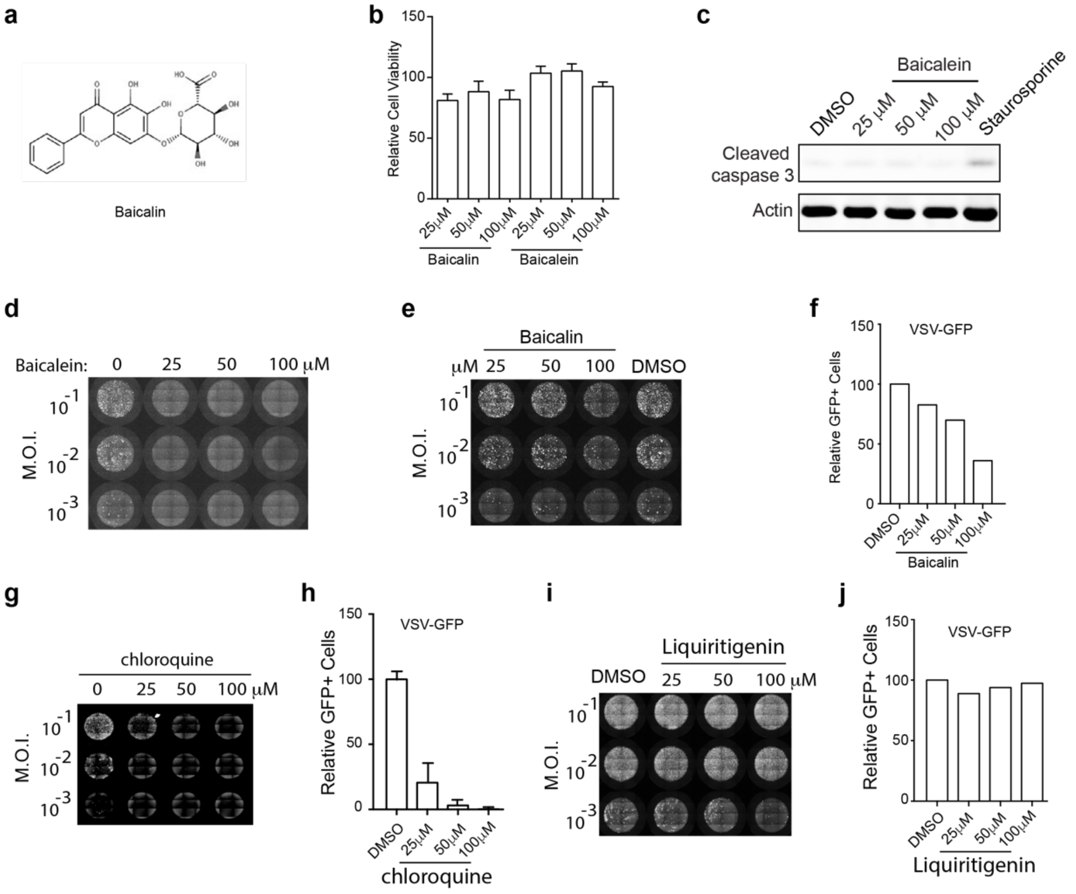

Fig. S1 Baicalein, Baicalin and chloroquine but not liquiritigenin inhibit VSV replication.

a. Baicalin structure. **b.** cell viability assay shows minimum toxic effect of Baicalin and Baicalein on Vero E6 cells under concentration of 100 μM or less. **c.** representative images of western blot show no detectable cleavaged caspase 3 after Baibalein treatment for 6 hours. Staurosporine (1μM) as a positive control. **d.** Representative image for VSV-GFP replication at different M.O.I. in the presence of increasing dose of Baicalein. **e.** Representative image for VSV-GFP replication at different M.O.I. in the presence of increasing dose of Baicalin. **f.** quantification of (**e**). **g.** Representative image for VSV-GFP replication at different M.O.I in the presence of increasing dose of chloroquine. **h.** quantification of (**g**). **i.** Representative image for VSV-GFP replication at different M.O.I in the presence of increasing dose of Liquiritigenin. **j.** quantification of (**i**). M.O.I.: multiplicity of infection.

Supplementary Figure 2

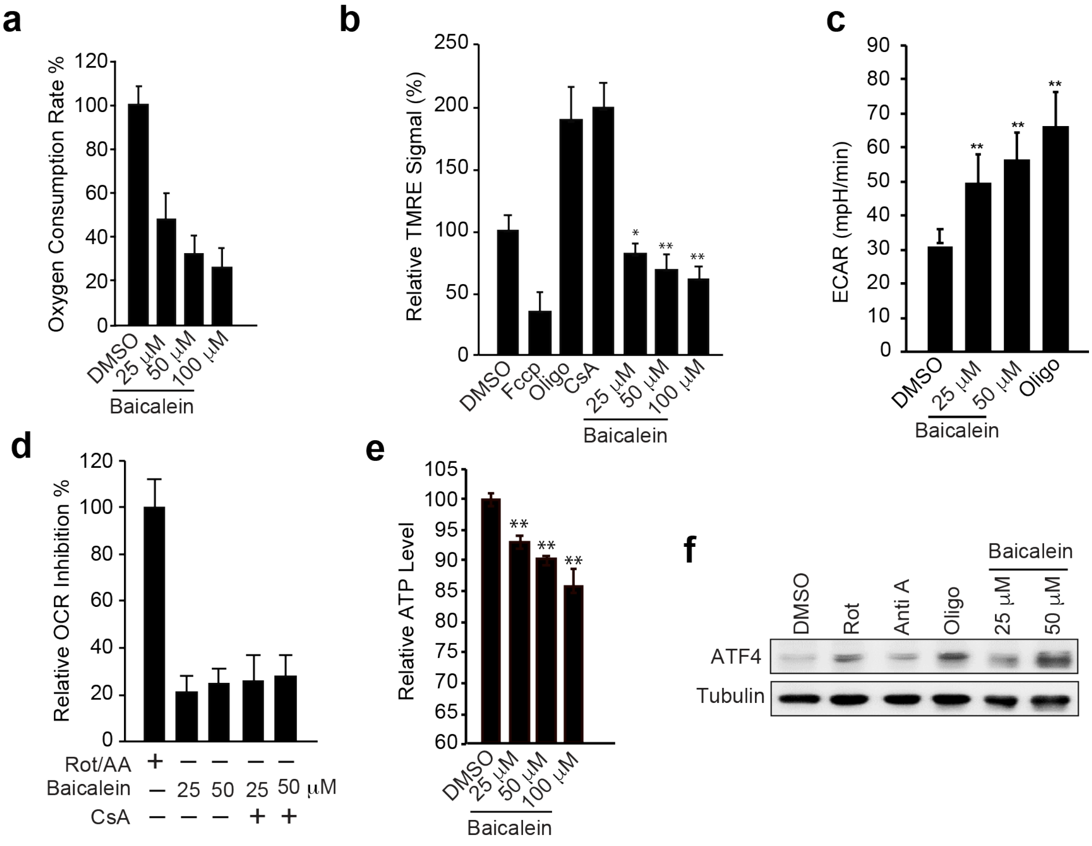

Fig. S2 Baicalein interferes mitochondrial oxidative phosphorylation

a. Oxygen consumption rate (OCR) assay shows dose dependent OCR inhibition by Baicalein in U937 cells. **b.** mitochondrial membrane potential measurement by TMRE in Vero E6 cells treated with mitochondrial uncoupler FCCP (1 μM), Oligomycin A (Oligo, 1 μM), Cyclosporine A (CsA, 1 μM), and increasing dose of Baicalein as indicated. **c.** Extracellular acidification rate (ECAR) measured in seahorse analyzer shows an instant upregulation of ECAR upon Baicalein treatment in VERO cells. **d.** quantification of relative OCR inhibition from seahorse analyzer data (as in Fig. 1**f**) with Vero E6 cells treated as indicated. **e.** ATP measurement by Cell Titer Glo® reveals Baicalein reduces Vero E6 cellular ATP level after 6 hours' treatment. **f.** Western blot shows upregulated ATF4 expression in Vero E6 cells treated as indicated. Rot: Rotenone (1 μM), Anti A: Antimycin A (1 μM), Oligo: Oligomycin A (1 μM).

Supplementary Figure 3

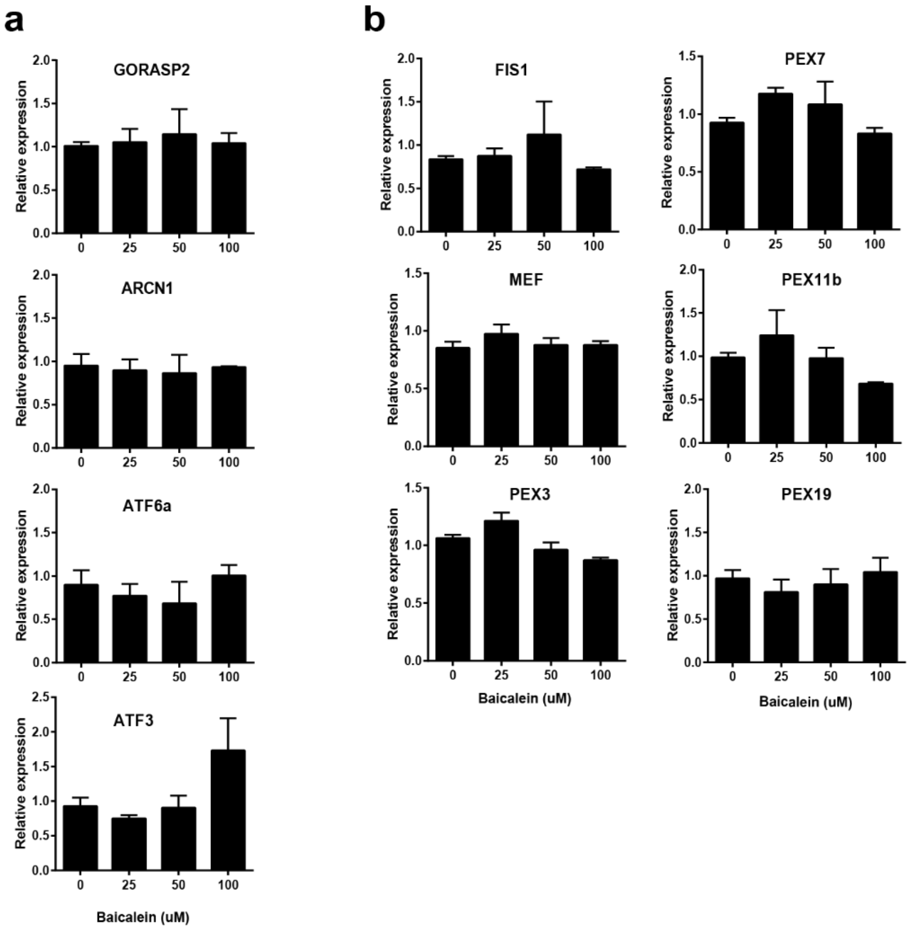

Fig. S3 Baicalein treatment does not affect function of ER and peroxisomes. **a**. RT-qPCR assays for transcripts of ER stress genes (GORASP2, ARCN1, ATF6a, and ATF3) indicates Baicalein treatment shows minimum effect on ER function. **b**. RT-qPCR assays for transcripts of genes (GORASP2, ARCN1, ATF6a, and ATF3) critically involved in peroxisome biogenesis indicate Baicalein treatment shows minimum effect on peroxisome function.

Supplementary Figure 4

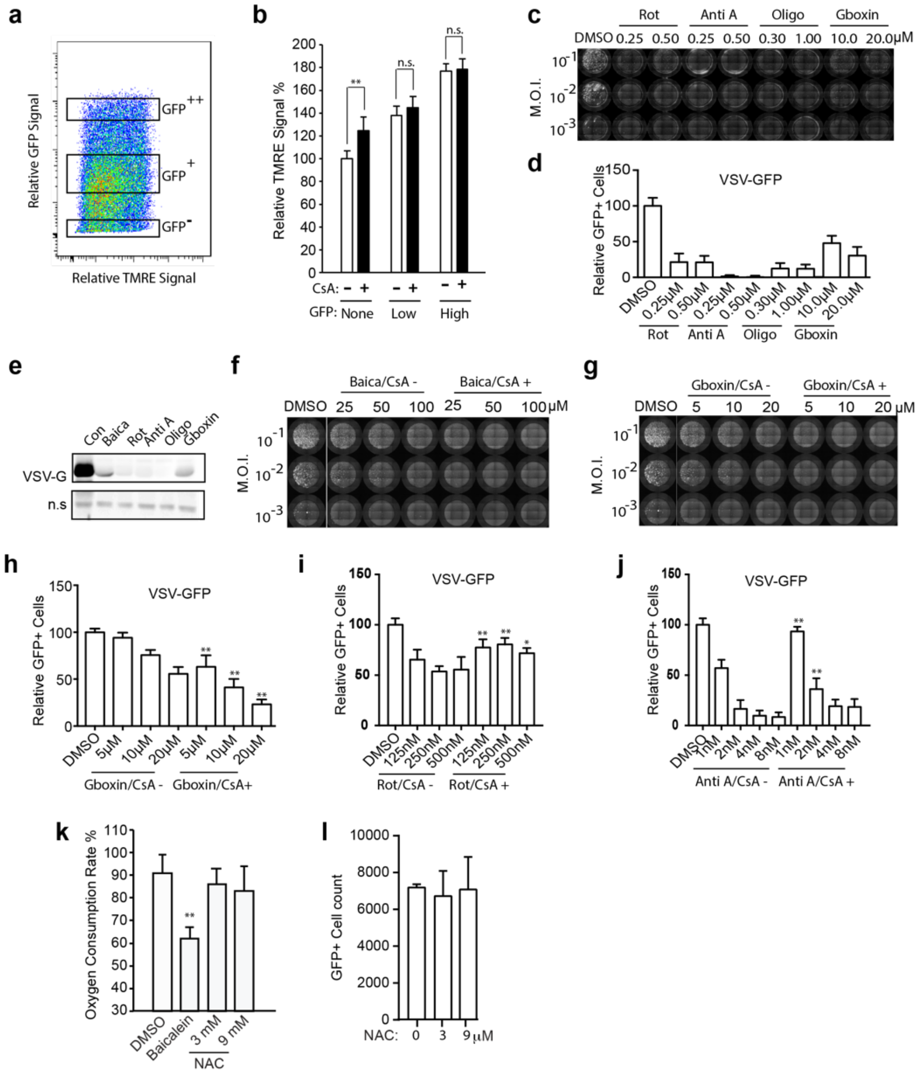

Fig. S4 VSV blunts mPTP and relies on mitochondrial OXPHOS for replication

a. Flow cytometry image for TMRE signal indicates increased mitochondrial membrane potential by VSV-GFP infection in Vero E6 cells. **b.** quantification of flow cytometry data for TMRE signal for mitochondrial membrane potential of VSV-GFP infected HEK293 cells treated with CsA or not. **c.** Representative image for VSV-GFP replication at different M.O.I in the presence of Rotenone (Rot), Antimycin A (Anti A), Oligomycin A (Oligo), and Gboxin. **d.** quantification of (**c**). **e.** Western blot for VSV-G protein in infected Vero E6 cells treated with reagents as indicated. **f.** Representative image for VSV-GFP replication at different M.O.I in the increasing dose of Baicalein (Baica) with or without mPTP inhibitor, CsA. **g.** Representative image for VSV-GFP replication at different M.O.I. in the presence of increasing dose of Gboxin with or without mPTP inhibitor CsA. **h.** quantification of (**g**). **i.** quantification of VSV-GFP replication at different M.O.I. in the increasing dose of Rotenone with or without mPTP inhibitor, CsA. **j.** quantification of VSV-GFP replication at different M.O.I. in

the increasing dose of Antimycin A with or without mPTP inhibitor CsA. **k**. Graph shows quantification of OCR of Vero E6 cells treated with reagents as indicated. **l.** quantification of GFP+ Vero E6 cells after infection shows treatment of ROS scavenger NAC does not affect VSV replication. * $p < 0.05$, ** $p < 0.01$. Rot: Rotenone, Anti A: Antimycin A, Oligo: Oligomycin A, Baica: Baicalein. M.O.I: multiplicity of infection.

Supplementary Figure 5

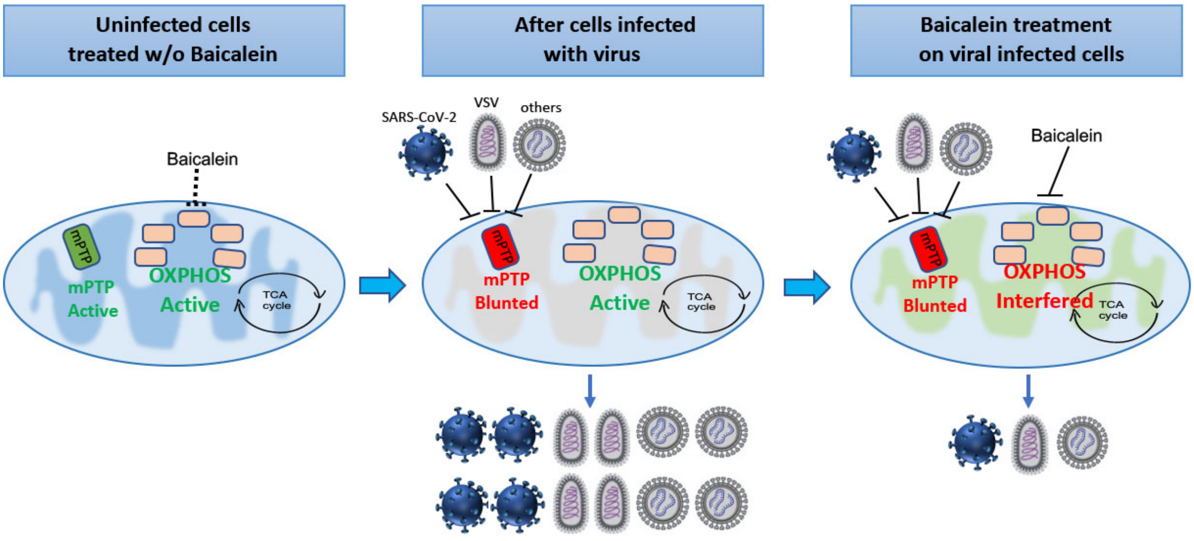

Fig. S5 Model for Baicalein mediated OXPHOS inhibition and viral suppression.

Uninfected cells have active mitochondrial mPTPs, which prevents Baicalein inhibition of mitochondrial OXPHOS activity. Viral infection (such as infection of SARS-Cov-2, VSV and others) blunts mitochondrial mPTPs, and the blunted mPTPs then render Baicalein mediated OXPHOS inhibition, which in turn limits viral replication in host cells.

Reference:

1. Y. N. Zhang *et al.*, Infectious Chikungunya Virus (CHIKV) with a Complete Capsid Deletion: a New Approach for a CHIKV Vaccine. *J Virol* **93**, (2019).
